# Age as a Predictor of Overall Survival in Colorectal Cancer

**DOI:** 10.3390/diagnostics14222550

**Published:** 2024-11-14

**Authors:** Berenice Carbajal-López, Jossimar Coronel-Hernández, Marytere Herrera, Erika Ruiz-Garcia, Sayako M. Miyagui-Adame, Consuelo Diaz-Romero, Eduardo Osiris Madrigal-Santillán, Priscila Morales Esponda-Mendoza, Carlos Pérez-Plasencia, Germán Calderillo-Ruiz

**Affiliations:** 1 Unidad Funcional de gastroenterología, Instituto Nacional de Cancerología, Tlalpan 14080, Mexico; eb.carbajalopez@gmail.com (B.C.-L.); mherrera_84@hotmail.com (M.H.); drasayakomiyagui@gmail.com (S.M.M.-A.); onco_diazz28@yahoo.com.mx (C.D.-R.); 2 Laboratorio de Genómica, Instituto Nacional de Cancerología, Tlalpan 14080, Mexico; jossithunders@gmail.com; 3 Dirección de Docencia y Laboratorio de Medicina Traslacional, Instituto Nacional de Cancerología, Tlalpan 14080, Mexico; betzabe100@yahoo.com.mx; 4 Laboratorio de Medicina de la Conservación, Escuela Superior de Medicina, Instituto Politécnico Nacional, Ciudad de Mexico 11340, Mexico; eomsmx@yahoo.com.mx; 5 Faculty of Medicine, Universidad Nacional Autónoma de México, Av. Universidad 3004, Copilco Universidad, Coyoacán, Ciudad de Mexico 04510, Mexico; priscila05.pm@gmail.com

**Keywords:** young, colorectal cancer, prognosis, overall survival

## Abstract

Background: The diagnosis of colorectal cancer (CRC) at early ages has become a challenging trend for oncology due to high rates of mortality worldwide. The correlation of clinical features with young-age prognosis in CRC remains unclear. Therefore, we aimed to describe the clinicopathological features and their impact on the overall survival of young Mexican adults diagnosed with CRC treated in the National Cancer Institute. Methods: This was a retrospective, observational study. The included patients were treated at the National Cancer Institute between 2004 and 2020. The statistical analyses comprised the X^2^ and *t* tests, Kaplan–Meier, log rank, and Cox regression. Statistical significances were assessed when *p* was bilaterally < 0.05. Results: A total of 3652 patients diagnosed with CRC attended the National Cancer Institute. Cases of early onset of CRC increased over the 16 years under study, with significant differences between the median age, from 57 in 2004 to 55 years old in 2020 (F = 5.49; gl: 12 *p* = 0.019). For this analysis, the population was divided in three groups: young (≤30 years), adults (31–70), and elderly (>70). The young population was mostly composed of men (62%; (*n* = 63), (*p* = 0.020), with high rates of metastatic disease (44%) (*p* = 0.001) and right-side tumors (57%), (*p* = 0.046), and with 44% with a moderate grade (*p* = 0.750). According to the overall survival (OS) analysis, the median OS was 29 months for young, versus 170 months for adult and 56 months for elderly patients (*p* <0.001, HR 1.53, 95% CI 1.11–2.10). A sub-analysis was performed considering only patients with metastatic disease. The median OS was 12 months for young, versus 17 and 9 months for adults and elderly (*p* = 0.08, HR 1.27, 95% CI 1.02–1.46). Conclusions: CRC diagnosis in the young population is increasing due unhealthy lifestyle habits and lack of screening. This population have clinical features of bad prognosis, such as left side, poor grade differentiation, and metastatic disease, precluding prognosis and OS.

## 1. Introduction

Colorectal cancer (CRC) is one of the main global health problems due to the increasing rates of incidence and mortality [1]. Currently, CRC is the third most common type of cancer and the second greatest cause of mortality worldwide [1,2]. Furthermore, the annual percentage of patients with an early onset of this disease has been rising within the past decades, with CRC being diagnosed in young adults. The incidence reported in this population was 5.5% in 2015. Nevertheless, it is expected to rise to 11% by 2030 [3,4]. Therefore, epidemiologic studies have aimed to study the possible causes of the high incidence of CRC in young populations, describing the relevance of family history, hereditary cancer syndromes, lifestyle, and environmental factors [5,6]. In addition, the lack of screening, poor knowledge of the disease among first-contact physicians, undervaluation or absence of symptoms, reluctance, and inaccessible healthcare services, preclude the accuracy of diagnosis of CRC in young people [1,2]. Furthermore, CRC in this population occurs mainly in the distal colon and rectum, often compromising regional lymph nodes and leading to metastasis at the time of diagnosis, and it is associated with perineural and lymph vascular invasion, poorly differentiated histological subtypes, mucinous or signet ring cells, and synchronous or metachronous tumors. It is worth noting that the factors previously described are associated with bad prognosis in CRC, precluding response to treatment and overall survival [1,2,3,4,5,7]. Therefore, although younger patients with CRC tend to have fewer comorbidities, emerging studies have reported worse survival rates for these patients, which may reflect the aggressive characteristics of the disease.

Furthermore, young age has been associated with low rates of progression-free survival and worse side effects from chemotherapy regimens compared to older patients [8,9]. By contrast, other studies have reported a better prognosis for young populations despite the poor prognostic factors mentioned above [10,11]. The differences in survival rates have been associated with the design of studies, histopathological subtypes, and the clinical stages of the populations included [10,11]. Considering these disparities, age has become increasingly relevant when considering the clinical management of CRC patients [12,13]. Understanding the clinicopathological characteristics of CRC in young patients will generate strategies for prevention and timely detection. Thus, this study aims to describe the clinicopathological features and overall survival in a cohort of young colorectal cancer patients compared to adults and elderly patients.

## 2. Materials and Methods

Retrospective, observational study of colorectal adenocarcinoma (CRC) patients treated at the Gastrointestinal Department of the National Cancer Institute, Mexico. Trial registration: retrospective study No. 2021/046.

### 2.1. Selection Criteria

The inclusion criteria consisted of female and male patients >18 years old, diagnosed with colorectal adenocarcinoma, and treated at the National Cancer Institute, Mexico, between January 2004 and December 2022. Exclusion criteria included incomplete clinical records, lack of three months of follow-up, and abandonment.

### 2.2. Clinical Data Collection

Clinical data included age, gender, tumor size, TNM stage (defined by the Eighth American Joint Committee on Cancer TNM system), histological differentiation, laterality, metastasis site, surgical procedure, chemotherapy treatment, radiotherapy, and at least three-month follow-up status.

### 2.3. Statistical Analysis

Patients were classified into three groups, young (≤30 years), adults (31–70 years), and elderly (>70 years), for the analysis. Statistical analysis was performed using SPSS v. 24 software (New York, NY, USA). Continuous variables were expressed as mean ± standard deviation values or as median and range (minimum and maximum) values. Categorical variables were expressed as percentages. Statistical comparisons among groups were performed using the *t*-test when data were normally distributed; otherwise, the Mann–Whitney U test was performed.

### 2.4. Overall Survival Analysis

Overall survival (OS) was defined as the time from diagnosis of CRC to death from any cause. Overall survival rates were calculated using the Kaplan–Meier method. Cox regression was performed for univariate and multivariate analyses to identify variable predictors of OS. Statistically significant difference was assessed when *p* <0.05 bilaterally.

## 3. Results

### 3.1. Young Patients with Colorectal Cancer Have More Clinical Features of Bad Prognosis

This study included 3652 patients diagnosed with colorectal adenocarcinoma at the Gastrointestinal Department at the National Cancer Institute, Mexico, from 2004 to 2022. For the analysis, the patients were classified into three groups: young, aged 30 years and younger (5%, *n* = 194 patients), adults, aged 31 to 70 years (75%, *n* = 2734 patients), and elderly, over 70 years of age (20%, *n* = 724 patients). The young patients were mostly male (62%, *n* = 120), while the adult and elderly groups reported proportions of male gender of 50% and 53%, respectively (*n* = 0.020). Regarding the laterality, a higher prevalence of right-sided CRC was observed in the young (57%, *n* = 111) and adult (57%, *n* = 1558) patients than in the elderly group (43%, *n* = 311) (*p* = 0.040). Regarding the histological characterization, the most prevalent subtype in the young patients was mucinous carcinoma (15%, *n* = 29), while this subtype was observed less frequently in the adults (8%, *n* = 219) and elderly (9%, *n* = 65) (*p* = 0.079). Additionally, the signet ring cell pattern was more prevalent among the young population (13%, *n* = 25), compared with the 3% reported in both the adult and elderly groups of patients (*p* <0.001). Moreover, regarding the grade of differentiation, the young patients reported a higher prevalence of poor differentiation (45%, *n* = 88), followed by moderately differentiated (44%, *n* = 85) and well differentiated (9%, *n* = 17), and with only 1% for undifferentiated and not classified. According to the clinical stage, the I–III stage was more prevalent in the adult (59%, *n* = 1613) and elderly (60%, *n* = 434) groups. Nevertheless, the prevalence of metastatic disease was 44% (*n* = 86) in the young population, whereas it was 36% (*n* = 994) in the adults, and 28% (*n* = 203) in the elderly group (*p* <0.001). Furthermore, the most frequent site of metastasis in the young patients was the peritoneum (58%, *n* = 54), in contrast to the adults and elderly, whose main site of metastasis was the liver (61%, *n* = 567, and 62%, *n* = 130, respectively) (*p* <0.001) (Table 1).

**Figure 1:**
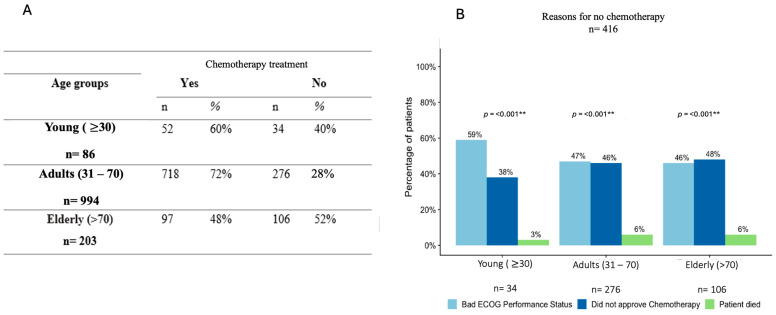
Chemotherapy treatment among metastatic patients. (**A**) describes the chemotherapy treatment among the groups of patients included. The highest prevalence of non-receipt of chemotherapy was among the young population. (**B**) describes the reasons for non-chemotherapy treatment. Bad ECOG (ECOG ≥ 3), was the main reason for non-eligibility for treatment in young patients. Statistical difference *p* < 0.005 (**).

On the other hand, the treatment for patients with metastatic disease (*n* = 1283) was chemotherapy, but only 60% (*n* = 52) of young, 72% (*n* = 718) of adult, and 48% (*n* = 97) of elderly patients received pharmacological treatment (Figure 1A). The reasons for non-eligibility for chemotherapy treatment differed between the groups: for the young patients with no chemotherapy (*n* = 34), 59% (*n* = 20) reported bad ECOG performance status (>2), another 38% (*n* = 13) did not accept chemotherapy treatment, and the remaining 3% (*n* = 1) died before eligibility (*p* <0.001) (Figure 1B).

**Figure 2:**
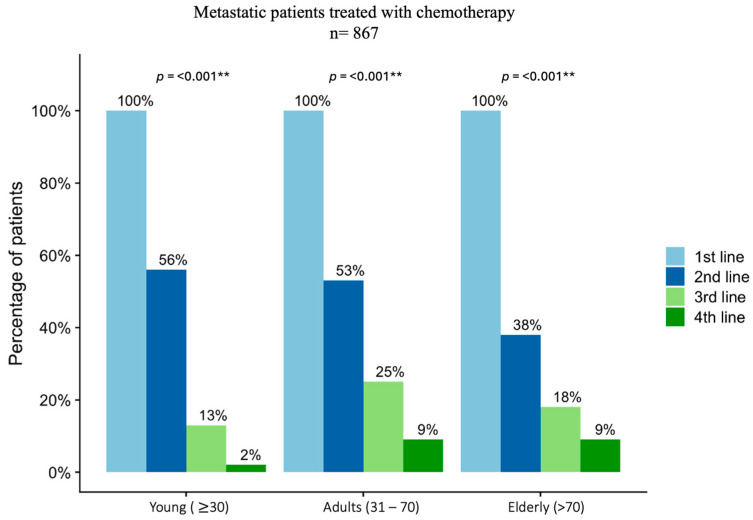
Lines of treatment for patients with metastatic disease. Differences were observed in eligibility of patients receiving multiple lines of treatment for colorectal metastatic disease. Statistical difference *p* < 0.005 (**).

On the other hand, for the patients who received chemotherapy (*n* = 867), the lines of treatment diverged when they were compared between the groups, especially when considering reaching multiple lines of treatment as the third and fourth line. Specifically, only 13% (*n* = 7) and 2% (*n* = 1) of the young patients presented clinical eligibility criteria for these lines of systemic treatment. Moreover, 25% (*n* = 182) and 18% (*n* = 17) of the adults and elderly, respectively, received third-line chemotherapy, while 9% (*n* = 61; *n* = 9) received the fourth line of treatment in both groups (*p* <0.001) (Figure 2).

### 3.2. Young Patients Have a Worse Prognosis for Overall Survival

**Figure 3:**
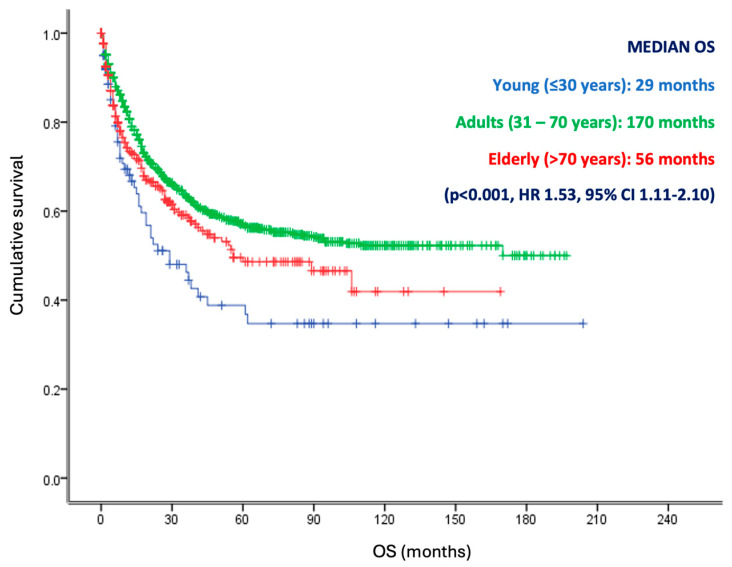
Overall survival of the global population according to age groups. Kaplan Meier curve that represent the differences in survival for the groups included, young (<30 years), adults (31–70 years), and elderly (>70 years). Young patients displayed a worse overall survival compared with adults and elderly patients. *p* <0.005.

**Figure 4:**
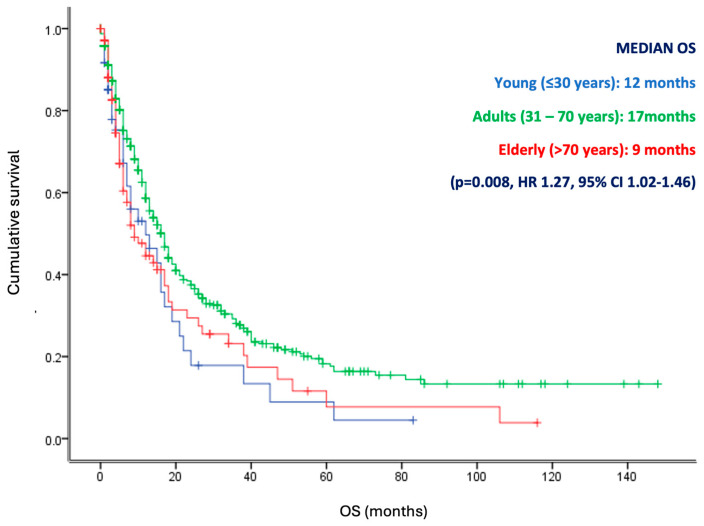
Overall survival of metastatic patients according to age groups. Kaplan Meier curve that represents the differences in survival for the groups included, young (<30 years), adults (31–70 years), and elderly (>70 years) in metastatic patients. Elderly and young patients displayed a worse overall survival compared with adult patients. *p* <0.005.

**Figure 5:**
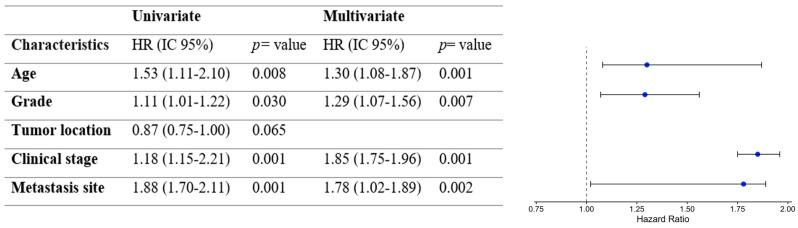
Cox proportional hazards for CRC patients treated at the National Cancer Institute. Clinicopathological features, age, grade, clinical stage, and metastasis site remained predictive factors of overall survival. *p* <0.005.

According to the overall survival analysis, the median OS for young patients was 29 months, compared to 170 months for adults, and 56 months for the elderly group of patients (*p* <0.001, HR 1.53, 95% CI 1.11–2.10) (Figure 3). Subsequently, a sub-analysis was performed by selecting patients with metastatic disease. In this analysis, we observed that the adult patients had a higher median OS (17 months) versus the young and elderly patients (12 months and 9 months) (*p* = 0.008, HR 1.27, 95% CI 1.02–1.46) (Figure 4). Furthermore, according to the univariate analysis, the age (HR: 1.53; *p*: 0.008), grade (HR: 1.11; *p*: 0.030), clinical stage (HR: 1.18; *p*: 0.001), and metastasis site (HR: 1.88; *p*: 0.001) were statistically significant. Additionally, when performing the multivariate analysis, only age (HR: 1.30; *p*: 0.001), grade (HR: 1.29; *p*: 0.007), clinical stage (HR: 1.85; *p*: 0.001), and metastasis site (HR: 1.78; *p*: 0.002) remained as independent predictors for OS (Figure 5). Therefore, age remains a significant predictor of overall survival, as well as grade, clinical stage, and metastasis site.

## 4. Discussion

This study described the clinicopathological characteristics and the overall survival (OS) rates of young Mexican adults diagnosed with CRC and treated at the National Cancer Institute. Although CRC incidence and mortality have been observed in the population aged 50 years and older, the diagnosis of colorectal cancer has recently increased in younger adults (<40 y.o.) [14]. Nevertheless, the age used to determine a “young population” remains controversial due to the heterogeneity of the ages and criteria reported in several countries. For example, in the US, young-CRC cases are reported between 40 and 49 years old [4]. Similarly, in countries such as Canada, Australia, and the United Kingdom (UK), young-CRC cases have been reported in adults younger than 50 years. The same scenario applies in Asia, where the “young population” with CRC has been described as individuals <50 years old [4,15]. However, in developing countries, age ranges are significantly different, including Brazil (18–39 y.o.) and Peru (20–49 y.o.) [10]. In our institution, the incidence of CRC is rising rapidly in the young population (<30 y.o.). For this reason, to evaluate the prevalence of young-CRC in our population and their clinicopathological features, we divided our population into three different groups: (1) young, (2) adult, and (3) elderly. The prevalence of male patients in the young population was high, which has previously been correlated with a bad prognostic outcome for CRC [3,4,6,16]. Furthermore, laterality has been described as a potential biomarker for worse outcomes in CRC patients, specifically right-side tumors, which show more aggressive behavior and poor overall survival rates [17]. Left-side laterality prevalence has previously been observed in young populations in other studies with significant differences (66% left-side versus 34% right-side tumors) [15,16,18,19,20]. Nevertheless, in our study, right-side laterality was the most prevalent anatomical subsite among young patients. Additionally, when comparing histopathological features, young patients showed a predominance of mucinous, poorly differentiated, and signet ring cell tumors, histopathological features previously associated with a bad prognosis and outcome [5,12]. Moreover, young patients are often diagnosed at advanced stages of the disease (36.5% and 25.3% stage III and IV, respectively) [2,5,12,14,21]. Similarly, in the present study, the young population showed a higher prevalence of metastatic disease (48%) compared to the adult (34%) and elderly patients (29%).

When comparing the sites of metastasis, the young population showed a higher prevalence of metastasis to the peritoneum, differing from adult and elderly patients, who reported the liver as the main site of metastasis. The control of liver metastasis has advanced significantly over the past decade, derived from the inclusion of metastasectomy and more effective schemes of chemotherapy [9]. Therefore, with the approach of a multidisciplinary team, CRC patients with liver metastasis can achieve 5-year survival rates higher than 30% [9,10,11]. On the other hand, peritoneum metastasis has been strongly correlated with a bad prognosis, due to the difficulties in controlling the disease at this body site [11,14]. Therefore, the young population included in our study had several characteristics associated with bad prognoses such as male gender, right-side laterality, and histopathological features such as mucinous carcinoma, poorly differentiated grade, and signet ring cell tumors. In addition, this population showed a higher prevalence of metastatic disease, mostly with metastasis to the peritoneum, highlighting the high rates of bad ECOG performance status (>2), which precludes clinical eligibility criteria for receiving systemic treatment. These findings are similar to those described in the literature, in which the young population has demonstrated similar features of bad prognosis, including advanced ECOG and metastatic disease, which lead these patients to receive best supportive care after the second line of treatment [7,8]. Considering the implications of these clinicopathological features associated with poor overall survival, we conducted a Kaplan–Meier analysis comparing the age groups included. In this analysis, the young patients showed worse median overall survival (29 months) compared with the adults (170 months) and elderly patients (56 months). Moreover, due to the high rates of metastatic disease in young patients described in our study, a sub-analysis was performed concerning the age groups. In this analysis, the young patients with metastatic disease showed statistical differences, with a worse median overall survival rate when compared to the adults and elderly patients. Furthermore, in the multivariate analysis, age (<30 years old), grade (poor), clinical stage, (metastatic), and site of metastasis remained as independent predictors of overall survival. Our findings correlate with previous information demonstrating that the younger population has an increased risk of death (19%), with a more advanced stage of disease at diagnosis and bad clinicopathological features, like poorly differentiated tumors and mucinous and/or signet ring histology [16,17,18]. The main challenge for the management of CRC is the high rate of metastatic disease at the time of diagnosis. In the Latin American population, several factors lead to a delay in the diagnosis of CRC, including the absence of awareness campaigns about the prevention and early detection of CRC among the young population, lack of knowledge or updates on CRC symptoms and detection among first-contact medical oncologists, and insufficiency of access to medical care or lack of social and family support [16]. In general, patients attend consultations after a long period of symptoms, due to family history of CRC, or other reasons that allow accidental detection, often in advanced stages of the disease. Furthermore, the avoidance of risk factors is part of primary prevention. However, more research is still needed to establish the risk factors for CRC onset among the young population. Secondary prevention involves screening studies, such as stool tests, flexible sigmoidoscopy, and colonoscopy, often referred to by patients as “a too-invasive procedure”. Nevertheless, the age to start screening is still a topic of discussion. In 2018, the American Cancer Society (ACS) suggested starting screening in the average-risk population at age 45, while for the high-risk population, it has been proposed to start at age 40, or 10 years prior [2,3,5,13].

This is one of the pioneering studies that correlates age and CRC clinicopathological features in the Latin American population. However, it still has some limitations, mainly its retrospective nature. Additionally, the present study did not analyze associations with hereditary CRC syndromes, such as MUTYH-associated polyposis, familial adenomatous polyposis (FAP), hereditary nonpolyposis colorectal cancer (HNPCC or Lynch syndrome), inflammatory bowel disease, and driver mutations of CRC, including KRAS, N-RAS, and Wild Type. Thus, a further prospective analysis is required to evaluate possible genetic associations in Mexican young patients, due to the relevance of molecular characterization in personalized medicine and treatment decisions for young patients with CRC.

Moreover, this study demonstrates the increasing incidence of CRC among young Mexican patients, with several clinicopathological features previously correlated with a worse OS, such as male gender, right-side laterality, poor degree of differentiation, and high prevalence of metastatic disease. The high exposure to risk factors in young Mexican populations, like obesity, a sedentary lifestyle, and an unhealthy diet, along with lack of screening and late detection, impair the proper diagnosis of colorectal cancer in this population, perpetuating bad prognoses for young patients. Therefore, a significant effort is required to achieve better outcomes for young Mexican patients, which includes primary and secondary prevention, an increase in awareness, and early screening in the high-risk population.

## 5. Conclusions

CRC diagnosis in the young population is increasing, and potential risk factors, along with the poor prognostic histopathologic features mentioned above, increase the risk of death in this population. Therefore, a prospective study that overcomes the limitations described is needed to answer the remaining questions related to the prognosis in Latin American early CRC. Furthermore, a global effort is required by the general population, first-contact physicians, and government strategies to raise awareness, to improve the early detection of this disease.

## Figures and Tables

**Table 1:** Clinicopathological features of patients with CRC according to age, treated at National Cancer Institute. Differences in clinicopathological features, gender, and histology differentiation grade. Clinical stage and site of metastasis were observed between the groups. Statistical difference *p* < 0.05 (**).

Characteristics	Groups Included (*n* = 3652)			*p* = Value
	Young (≤30 years) *n* = 194	Adults (31–70 years) *n* = 2734	Elderly (>70 years) *n* = 724	
Sex				0.020 **
Male	120 (62%)	1367 (50%)	384 (53%)	
Female	74 (38%)	1367 (50%)	340 (47%)	
Tumor location				0.040 **
Left side	83 (43%)	1176 (43%)	413 (57%)	
Right side	111 (57%)	1558 (57%)	311 (43%)	
Histology				
Mucinous	29 (15%)	219 (8%)	65 (9%)	0.079
Signet ring cells	25 (13%)	82 (3%)	22 (3%)	<0.001 **
Grade				0.750
Well differentiated	17 (9%)	383 (14%)	109 (15%)	
Moderately differentiated	85 (44%)	1586 (58%)	420 (58%)	
Poor differentiated	88 (45%)	656 (24%)	166 (23%)	
Undifferentiated	2 (1%)	27 (1%)	7 (1%)	
Not classficiable	2 (1%)	82 (3%)	22 (3%)	
Clinical stage				<0.001 **
Clinical stage I–III	87 (45%)	1613 (59%)	434 (60%)	
Locally advanced and unresecable	21 (11%)	137 (5%)	87 (12%)	
Metastatic disease	86 (44%)	994 (36%)	203 (28%)	
Metastasis site				<0.001 **
Liver	34 (40%)	601 (61%)	124 (62%)	
Lung	5 (6%)	121 (12%)	37 (18%)	
Peritoneal carcinomatosis	54 (58%)	125 (13%)	17 (8%)	
Retroperitoneum (nodes)	3 (4%)	88 (9%)	12 (6%)	

## Data Availability

The data presented in this study are available on request from the corresponding author. The data are not publicly available due to patient information included is protected and proprietary to the National Cancer Institute.
